# Digital Screen Time and Mental Health Difficulties Among Preschool Children in Western China: Cross-Sectional Study

**DOI:** 10.2196/99762

**Published:** 2026-08-17

**Authors:** Jia Wang, Hongli Sun, Mingyue Duan, Xi Zhang

**Affiliations:** 1 School of Politics, Law & Public Administration, Yan’an University, Yan’an, Shaanxi, China; 2 Department of Sociology, School of Humanities and Social Sciences, Xi'an Jiaotong University, Xi’an, Shaanxi, China; 3 Shaanxi Institute for Pediatric Diseases, Xi’an Key Laboratory of Children’s Health and Diseases, Xi’an Children’s Hospital (Affiliated Children’s Hospital of Xi’an Jiaotong University), Xi’an, Shaanxi, China; 4 Department of Clinical Laboratory, Xi'an Children's Hospital, Affiliated Children's Hospital of Xi'an Jiaotong University, National Regional Children's Medical Center (Northwest), No. 69 Xijuyuan Lane, Lianhu District, Xi’an, Shaanxi, 710003, China, 86 87692102

**Keywords:** screen time, preschool children, mental health, mediation analysis, parenting

## Abstract

**Background:**

With rising digital media use among young children, concerns have grown regarding its impact on early socioemotional development. However, evidence regarding the dose-response relationship between digital screen time and children’s mental health, as well as the potential indirect statistical association with family factors, remains limited.

**Objective:**

The study aimed to explore the nonlinear association between screen time and mental health outcomes in preschool children, identify a potential threshold, and examine the indirect statistical association of parental discipline in this relationship.

**Methods:**

This cross-sectional study included 21,366 parent-child dyads from Western China. Participants were recruited through stratified cluster sampling from 189 public kindergartens across 13 districts between February 28 and March 5, 2025. Caregivers of children aged 3 to 6 years completed questionnaires administered by trained kindergarten teachers, reporting children’s average daily screen time (weekdays/weekends) over the preceding month. Children’s mental health was assessed using the Strengths and Difficulties Questionnaire. Generalized additive models and piecewise logistic regression identified nonlinear patterns and thresholds. Mediation analysis quantified the indirect statistical association of parental discipline, and moderation analyses examined interaction effects. Sensitivity analyses included threshold dichotomization, exclusion of extreme values, E-value calculation, multiple imputation for missing data, and reverse-direction mediation.

**Results:**

A significant nonlinear association was found, with a saturating dose-response pattern. A 2-piecewise logistic regression reparameterization yielded a policy-interpretable summary of approximately 70 minutes per day (95% CI 66‐75) for elevated clinical risk. Below this summary estimate, each additional hour of screen time was associated with 65% greater odds of total difficulties (odds ratio 1.65, 95% CI 1.46‐1.85); above the summary estimate, the association was greatly attenuated (odds ratio 1.07, 95% CI 1.01‐1.13). However, the smooth generalized additive models fit marginally better (ΔAkaike information criterion=−6.78), indicating that the association is better characterized as a graded, saturating pattern rather than a sharply defined break point. Weekday screen time showed a stronger dose-response pattern than weekend screen time. Subgroup analyses suggested tentative moderation by the number of children and household registration (*hukou*, the birth-based urban vs rural classification that determines access to locally funded services such as education and health care), although these effects were not significant after false discovery rate correction. Parental discipline accounted for 20.5% of the association with total difficulties (95% CI 15.8%‐26.2%), with similar patterns for externalizing (21.2%, 95% CI 15.7%‐28.4%) and internalizing problems (23.6%, 95% CI 17.2%‐33.5%). Multiple sensitivity analyses confirmed robustness of the findings.

**Conclusions:**

Daily digital screen time shows a nonlinear association with preschool children’s mental health outcomes. Reducing weekday screen exposure and optimizing parental discipline represent promising intervention targets, based on cross-sectionally derived correlational mediation patterns. These findings offer locally relevant, evidence-based benchmarks for screen-time guidance and highlight modifiable parenting factors for early childhood digital health interventions.

## Introduction

Children’s mental health problems, including internalizing and externalizing symptoms, are prevalent and associated with long-term developmental impairments [1,2]. During the preschool years, early manifestations of such difficulties can disrupt social, emotional, and cognitive trajectories, underscoring the need for timely identification and intervention [3]. Accumulating evidence indicates that the preschool period is a critical window for shaping mental health outcomes, as neural and behavioral plasticity is high and early symptoms often predict persistent psychopathology [4,5]. In Western China, as in many regions, understanding the modifiable determinants of early mental health problems remains a public health priority [2].

The digital age has witnessed a dramatic rise in screen use among children and adolescents, who increasingly rely on digital devices such as televisions, computers, smartphones, tablets, and video games [6,7]. Even prior to the COVID-19 pandemic, screen time was steadily rising, and it surged dramatically during the public health crisis [8,9]. Notably, this elevated usage has persisted beyond the acute phases of the pandemic [10], suggesting a sustained shift in media consumption habits. Statistics indicate that in many countries worldwide, children’s average daily screen time has exceeded recommended guidelines [11]. As screens have become deeply embedded in daily life, growing concern has emerged regarding their potential implications for children’s health and development. Excessive screen exposure is increasingly recognized as a multifaceted risk factor for children’s health. Accumulating evidence indicates an association between excessive screen exposure and adverse physical health outcomes, including myopia, elevated risks of obesity, and adverse cardiovascular markers such as increased blood pressure [12-14]. Beyond physical health, the detrimental effects of screen time extend to children’s mental and behavioral well-being, an equally concerning domain. A growing body of research has consistently reported associations between higher screen use and poorer mental well-being in children and adolescents, manifesting as increased depressive symptoms, anxiety, attentional difficulties, and conduct problems [15-19], highlighting the need for a nuanced understanding of its role in children’s health.

As a key environmental factor affecting children’s mental health, parental discipline styles exert a profound and long-lasting impact on children’s socioemotional development, cognitive function, and mental health outcomes [20,21]. Different parental discipline behaviors show distinct associations with children’s mental health: harsh and aggressive discipline strategies, including psychological and physical aggression, are consistently linked to adverse child outcomes, such as increased aggressive behaviors, distraction, and elevated risk of emotional and behavioral problems [22,23]. Improper discipline, such as physical punishment, is associated with increased suicidal behaviors in adolescents [24]. Notably, harsh discipline in early childhood can even alter children’s cortical development, affecting the neural circuits related to social and sensorimotor functioning [25], and may increase reward sensitivity in adolescence, further influencing mental health trajectories [26]. In contrast, positive discipline approaches and techniques, such as verbal reasoning and time-out, are associated with lower odds of child aggression, higher levels of prosocial peer relations, and better emotion regulation and mental health in adulthood, contributing to positive socioemotional development [22,27]. These findings highlight that parental discipline is not only a core part of the family environment but also a critical intervention target for promoting children’s mental health.

Although the direct association between screen time and children’s mental health problems has been documented [15-17], most studies have focused on school-aged children and adolescents rather than preschool children, and the mechanisms underlying this relationship remain not fully understood. A growing body of research has moved beyond examining direct effects to explore potential indirect statistical linkages. Several factors have been identified as potential mediators in this association, including reduced sleep duration, diminished outdoor activity, and inadequate parental monitoring [28-30]. However, the role of parental discipline—an essential component of the family environment—has been inadequately addressed. We propose that parental discipline may serve as an indirect statistical association linking screen time to children’s mental health problems. Given that increased screen time may exacerbate parent-child conflicts and undermine effective limit-settings [31], it is plausible that parental discipline acts as an indirect statistical linkage. Specifically, prolonged screen exposure may provoke parental frustration or harsh disciplinary responses, which in turn contribute to poorer children’s mental health outcomes. Therefore, we hypothesized that parental discipline links between screen time and mental health outcomes in preschool children.

This cross-sectional study aimed to (1) examine the association between daily screen time and preschool children’s mental health outcomes (total difficulties, externalizing, and internalizing problems) in a sample from Western China, with a focus on identifying potential nonlinear patterns and threshold effects; and (2) quantify the extent of the indirect statistical association through parental discipline in the relationship between screen time and mental health.

## Method

### Study Design and Sample

This cross-sectional analysis was based on data from a large preschool cohort established in Western China via collaboration with local education authorities. To ensure the representativeness of the study population, a stratified cluster sampling method was adopted to randomly select 189 public kindergartens across 13 districts, with sampling proportions adjusted to match the distribution of preschool-aged children in each region. After obtaining cooperation consent from each sampled kindergarten’s management team, research staff coordinated with class teachers to distribute questionnaires to all families of children aged 3 to 6 years enrolled in the institution. Teachers distributed the questionnaires to children to take home, and primary caregivers (who provided at least 80% of daily care) completed the self-administered questionnaires offline before returning the completed forms to classroom teachers within 6 days. Every eligible child within each sampled kindergarten received an invitation to participate, with no random subsampling or selective exclusion of children. Before the formal survey, a small-scale pilot test was carried out to check the clarity of the questionnaire, and minor wording adjustments were made accordingly. Children were not present during questionnaire completion. Kindergarten teachers received basic training on the study purpose, questionnaire instructions, and privacy protection prior to the survey. Fieldwork was conducted from February 28 to March 5, 2025.

The original survey enrolled 25,017 child-caregiver dyads. After excluding 2408 nonparental caregivers, the sample comprised 22,609 parent-child dyads. A total of 1243 records were further excluded due to missing or implausible age values: parental age (n=517, 2.3%) and child age (n=726, 3.2%), while all key exposure and outcome variables were fully recorded. Complete-case analysis was performed on the final analytical sample of 21,366 dyads.

### Ethical Considerations

The study protocol was approved by the Medical Ethics Committee of Xi’an Jiaotong University Affiliated Children’s Hospital (approval number 20250225‐21). All procedures adhered to the principles of the Declaration of Helsinki, using anonymized datasets, and written informed consent was provided by all participating guardians. The study also followed the STROBE (Strengthening the Reporting of Observational Studies in Epidemiology) guidelines for cross-sectional studies (Checklist 1).

### Children’s Mental Health

Children’s mental health outcomes were assessed using the official simplified Chinese version of the Strengths and Difficulties Questionnaire (SDQ), a well-validated 25-item tool that has been proven to have good reliability and validity among Chinese children aged 3 to 17 years [32-34]. We defined elevated behavioral and emotional difficulties as total difficulty scores above 14, a cutoff value that has been well validated for Chinese children in previous local research [33,34]. The Cronbach α value of the total difficulties scale in our sample was 0.703, consistent with prior Chinese validation studies [35]. This study focused on 3 key outcome measures from the 5 subscales of the SDQ: total difficulties, externalizing problems, and internalizing problems.

These 3 measures were selected for the following reasons. The total difficulties score is the most commonly used global composite metric in children’s mental health epidemiology to evaluate overall psychological risk [34]. Externalizing and internalizing problems represent the 2 broad psychopathology domains most consistently linked to screen time and other digital environmental exposures in preschool children [36,37]. The peer problems and prosocial behavior subscales were not included in the present analysis, as peer difficulties are predominantly shaped by school-based social environments and show weaker associations with screen use, while prosocial behavior measures positive social strengths rather than adverse mental health outcomes—both of which are beyond the scope of this investigation [38].

Total difficulties: A composite measure (range: 0‐40) derived from 4 subscales, including emotional symptoms, conduct problems, hyperactivity/inattention, and peer problems, and each scored on a 3-point Likert scale ranging from 0 (“does not apply”) to 2 (“completely applies”). Five items (7, 11, 14, 21, and 25) were reverse-scored. A total difficulties score above 14 is defined as being at risk for mental health problems.

Corresponding subscale thresholds were defined as follows: emotional symptoms >3, conduct problems >2, hyperactivity/inattention >6, and peer problems >4. These subscales were further combined into 2 broad domains: externalizing symptoms, including conduct problems and hyperactivity/inattention, and internalizing symptoms based on the presence of emotional symptoms.

### Digital Screen-Time Exposure

In this study, digital screen time refers to the use of digital devices, including televisions, computers, tablets, and smartphones. Parents reported their child’s screen time on weekdays and weekends over the preceding month. A 1-month recall period was used, as it balances the need to capture typical screen use patterns against caregiver recall burden and is consistent with prior population-based studies of screen exposure in young children [39]. Responses were categorized into five groups: <30, 30 to 59, 60 to 119, 120 to 180, and >180 minutes per day. Average daily screen time was computed as (weekday min×5+weekend min×2)/7 and then converted to hours for continuous analyses.

### Parental Discipline

Dysfunctional parental discipline was assessed using the Parenting Scale, a multidimensional measure comprising 3 subscales: overreactivity, laxness, and verbosity. Responses were rated on a 7-point continuum scale; higher total scores indicate more dysfunctional parenting practices [40]. The Parenting Scale has been validated across diverse cultural settings [41,42]. In the current sample, the scale demonstrated excellent internal consistency (Cronbach α=0.926 in this study).

### Covariates

All covariates were selected before statistical analysis based on established associations with children’s mental health outcomes from the prior literature. These included child and primary parent demographics (age and gender), family structure (number of children and marital status) [43], health behaviors (smoking and alcohol use) [44], and socioeconomic factors (education, occupation, income, and household registration) [45-47].

### Statistical Analysis

Continuous variables were presented as mean (SD), and categorical variables as quantity (proportion). Between-group comparisons were performed using the Kruskal-Wallis *H* test for continuous measures and Fisher exact test for categorical measures. Pearson or Spearman correlation coefficients were calculated to examine pairwise associations among all study variables. Multicollinearity was checked using variance inflation factors, and all values were below 5, indicating no significant collinearity and supporting the inclusion of all covariates in regression models (Table S1 in Multimedia Appendix 1). To assess the potential influence of same-source common method bias, we performed the Harman single-factor test. All measured items were entered into an unrotated exploratory factor analysis. The first extracted factor accounted for 28.97% of the total item variance, well below the conventional 40% threshold, suggesting that common method bias was unlikely to substantially affect our findings. Nevertheless, this single-factor test is a necessary but not sufficient diagnostic for common method bias [48,49]. Residual same-source influences cannot be completely excluded, and findings should be interpreted with this limitation in mind.

Generalized additive models (GAMs) with a logit link were used to assess nonlinearity in the dose-response relationship, with a chi-square test for the smooth term. A 2-piecewise logistic regression model with a bootstrap-estimated break point (1000 resamples) was then applied to identify a threshold. The optimal cut point was selected by maximizing the log-likelihood, and its 95% CI was derived from the bootstrap distribution. The threshold model was compared with a linear model using a log-likelihood ratio test. This segmented model produces separate effect estimates on either side of the cut point, offering greater clinical interpretability. Odds ratios (ORs) were reported per 1-minute increase and converted to per 1-hour increase for clinical interpretability. To compare relative goodness-of-fit between the 2-piece threshold model and a smooth GAM, we calculated and contrasted the Akaike information criterion (AIC) for both nonlinear specifications. Finally, to mitigate information loss arising from dichotomizing SDQ scores into binary risk categories, we performed supplementary nonlinear analyses using continuous total difficulty scores as the outcome, including a GAM and 2-piece linear regression to capture symptom severity alongside the primary binary threshold results.

Multivariable logistic regression models were constructed to evaluate the independent associations of daily screen time (both as a continuous variable in hours and as a categorical variable) with children’s mental health outcomes, with results reported as crude and adjusted ORs and 95% CIs. Subgroup analyses and interaction tests were also performed to identify potential effect modification across sociodemographic subgroups. The subgroup variables included all covariates adjusted for in the main models. These variables were prespecified based on prior literature and examined systematically as potential effect modifiers. Interaction *P* values were adjusted for multiple comparisons using the false discovery rate (FDR) method.

A regression-based mediation analysis was conducted to assess whether parental discipline (overreactivity, laxness, and verbosity) statistically mediated the association between daily screen time and children’s mental health difficulties. In sequential regression models, we first examined the attenuation of effect sizes after adjusting for the potential mediator. We then estimated the direct and indirect effects using a bootstrap resampling approach with 1000 iterations to derive bias-corrected 95% CIs. The mediating proportion was calculated to quantify the relative contribution of the indirect effect. Statistical significance was defined as a 95% CI that did not include 0. All mediation models were fully adjusted for the identical set of covariates used in primary regression analyses to mitigate confounding bias in both the exposure-mediator and mediator-outcome pathways. Given the cross-sectional design, findings should be interpreted as statistical associations rather than definitive causal pathways, and the temporal ordering assumption could not be formally tested [50].

Several sensitivity analyses were performed to assess the robustness of the main findings. First, screen time was dichotomized at the identified threshold and reentered into the logistic regression models for all outcomes. Second, we excluded participants with extreme weekday screen time (≥180 min) to evaluate the influence of outliers. Third, the potential impact of unmeasured confounding was quantified using the E-value analysis [51]. E-values were calculated based on the risk ratio approximation, accounting for the nonrare outcome of total difficulties (a prevalence of about 18.6%). Fourth, we used multiple imputation by chained equations to evaluate the robustness of the results under missing data. Under the missing-at-random framework, we generated 5 imputed datasets, including all exposure indicators, mental health outcomes, and demographic covariates as predictors. The pooled regression estimates from the imputed data were then compared with the complete-case results. Fifth, to assess the robustness of the mediation results to the assumed direction of effects, we fit a reverse-direction mediation model in which children’s mental health problems were specified as the exposure, screen time as the outcome, and parental discipline as the mediator, adjusting for the same covariates as the main mediation analysis. All sensitivity analyses adjusted for the same covariates as the main models.

All analyses were performed on the full sample (N=21,366) using R software (version 4.5.2) and EmpowerStats (X&Y solution) [52], with statistical significance set at a 2-tailed *P*<.05.

## Results

### Participant Characteristics

Table 1 presents the baseline characteristics of the 21,366 participants by total difficulties score (≤14 vs >14). Children in the higher-scoring group (>14) had longer daily screen time (mean 77.33, SD 63.21 vs mean 64.95, SD 56.99 min), higher parental discipline scores (118.06 vs 111.33, indicating more dysfunctional parenting), and a higher proportion of boys (54.4% vs 51.2%). Their parents were younger (34.48 vs 34.81 y), the primary parent was more often male (28.8% vs 22.8%), and they had higher rates of smoking (18.7% vs 14.0%) and alcohol use (21.1% vs 15.4%). Socioeconomic disadvantages were also more prevalent in this group, including lower educational attainment (≤ junior high school: 35.3% vs 24.9%), lower annual family income (<30,000 CNY, 1 CNY=US $0.140 in 2025: 49.5% vs 38.3%), rural household registration (71.8% vs 65.5%), and lower employment rates (68.6% vs 74.3%). A clear dose-response relationship was observed between increasing screen time (both weekday and weekend) and higher total difficulties scores. The prevalence of externalizing and internalizing problems was 24.2% and 20.9%, respectively. Baseline characteristics stratified by these outcomes are shown in Table S2 in Multimedia Appendix 1, and those stratified by screen time are shown in Table S3 in Multimedia Appendix 1.

Correlations among all study variables are shown in Table S4 in Multimedia Appendix 1. Briefly, screen time was positively correlated with children’s mental health outcomes and parental discipline.

**Table 1.** Characteristics of participants by total difficulties in children (N=21,366)^a^.

Variables	Total sample (N=21,366)	Total difficulties	
		Normal, ≤14 (n**=**17,388)	At risk of mental health problems, >14 (n=3978)
Child age (y), mean (SD)	4.82 (0.89)	4.82 (0.88)	4.80 (0.90)
Primary parent age (y), mean (SD)	34.75 (4.56)	34.81 (4.53)	34.48 (4.68)
Parental discipline (PS), mean (SD)	112.58 (13.29)	111.33 (13.61)	118.06 (10.09)
Average daily screen time (min), mean (SD)	67.25 (58.40)	64.95 (56.99)	77.33 (63.21)
Child gender, n (%)			
Boys	11,062 (51.8)	8897 (51.2)	2165 (54.4)
Girls	10,304 (48.2)	8491 (48.8)	1813 (45.6)
Primary parent gender, n (%)			
Man	5108 (23.9)	3961 (22.8)	1147 (28.8)
Woman	16,258 (76.1)	13,427 (77.2)	2831 (71.2)
Smoking history, n (%)			
Yes	3179 (14.9)	2436 (14.0)	743 (18.7)
No	18,187 (85.1)	14,952 (86.0)	3235 (81.3)
Alcohol history, n (%)			
Yes	3508 (16.4)	2670 (15.4)	838 (21.1)
No	17,858 (83.6)	14,718 (84.6)	3140 (78.9)
Number of children, n (%)			
1	6148 (28.8)	4981 (28.7)	1167 (29.3)
2	12,844 (60.1)	10,527 (60.5)	2317 (58.3)
≥3	2374 (11.1)	1880 (10.8)	494 (12.4)
Marital status, n (%)			
Married/cohabitating	20,761 (97.2)	16,939 (97.4)	3822 (96.1)
Widowed/divorced/separated	605 (2.8)	449 (2.6)	156 (3.9)
Employment status, n (%)			
Working	15,642 (73.2)	12,912 (74.3)	2730 (68.6)
Not working	5724 (26.8)	4476 (25.7)	1248 (31.4)
Education level, n (%)			
≤ Junior high school	5741 (26.9)	4337 (24.9)	1404 (35.3)
High school diploma	4093 (19.2)	3353 (19.3)	740 (18.6)
Junior college	5383 (25.2)	4447 (25.6)	936 (23.5)
≥ Undergraduate degree	6149 (28.8)	5251 (30.2)	898 (22.6)
Annual family income (CNY)^b^, n (%)			
<30,000	8619 (40.3)	6652 (38.3)	1967 (49.5)
30,000-99,999	8619 (40.3)	7168 (41.2)	1451 (36.5)
≥100,000	4128 (19.3)	3568 (20.5)	560 (14.1)
Household registration of children, n (%)			
Urban	7116 (33.3)	5993 (34.5)	1123 (28.2)
Rural	14,250 (66.7)	11,395 (65.5)	2855 (71.8)
Daily screen time (min), n (%)			
Weekdays			
<30	7272 (34.0)	6204 (35.7)	1068 (26.9)
30-59	7960 (37.3)	6521 (37.5)	1439 (36.2)
60-119	2813 (13.2)	2164 (12.5)	649 (16.3)
120-179	908 (4.3)	680 (3.9)	228 (5.7)
≥180	2413 (11.3)	1819 (10.5)	594 (14.9)
Weekends			
<30	4479 (21.0)	3693 (21.2)	786 (19.8)
30-59	7099 (33.2)	5911 (34.0)	1188 (29.9)
60-119	4442 (20.8)	3658 (21.0)	784 (19.7)
120-179	1937 (9.1)	1527 (8.8)	410 (10.3)
≥180	3409 (16.0)	2599 (15.0)	810 (20.4)

a Continuous variables are expressed as mean (SD) and categorical variables are expressed as frequency (%).

b All household income values are presented in Chinese Yuan (CNY). CNY values were converted to US dollars using the average exchange rate of 1 CNY=US $0.140 in 2025.

### Nonlinear Relationship and Threshold Effect of Screen Time on Mental Health Outcomes

**Figure 1.**
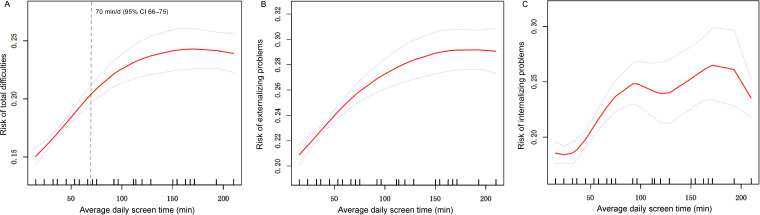
Nonlinear associations between average daily screen time and children’s mental health outcomes: (A) total difficulties, (B) externalizing problems, and (C) internalizing problems. The red solid line represents the fitted curve, and the blue dashed lines represent the 95% CIs. The vertical line in (A) indicates the threshold (approximately 70 min/d; 95% CI 66‐75) identified by the 2-piecewise logistic regression for total difficulties; threshold analysis was not performed for externalizing or internalizing problems.

GAMs were used to explore nonlinear associations between average daily screen time and mental health outcomes (Figure 1; model fit statistics are shown in Table S5 in Multimedia Appendix 1). For total difficulties and externalizing problems, significant monotonically increasing associations were observed. Risk rose rapidly at lower levels of screen time and gradually plateaued at around 100 minutes, with similar patterns between the 2 outcomes. For internalizing problems, the association was nonlinear and fluctuating, without a clear monotonic trend.

To quantify the nonlinearity in total difficulties, piecewise logistic regression was performed, which identified a significant threshold effect at approximately 70 minutes per day (95% CI 66‐75; Table S6 in Multimedia Appendix 1). Below this threshold, each additional hour of screen time was associated with a 65% higher risk of total difficulties (OR 1.65, 95% CI 1.46‐1.85). Above the threshold, the association remained statistically significant but was greatly attenuated: each additional hour of screen time corresponded to a 7% increase in odds (OR 1.07, 95% CI 1.01‐1.13). This pattern is consistent with the nonlinear curve shown in Figure 1, where the marginal increase in risk diminishes and plateaus at higher screen-time levels.

We assessed model performance via AIC comparison, yielding a ΔAIC=−6.78. Although the GAM exhibited marginally better statistical fit, the absolute difference between models was modest (|ΔAIC|<10). We retained the 2-piecewise framework for the primary results, as it generates an interpretable threshold (approximately 70 min/d) for clinical and public health applications. Full AIC values are provided in Table S7 in Multimedia Appendix 1. A likelihood ratio test further confirmed a significant threshold effect relative to simple linear regression (*P*<.001).

To mitigate information loss introduced by the binary SDQ classification, supplementary nonlinear models were constructed with continuous total difficulty scores as the outcome (Figure S1; model fit statistics are shown in Table S8 in Multimedia Appendix 1). Consistent with the primary binary outcome results, the continuous analysis revealed a nonlinear association between screen time and total difficulty scores. Two-piece linear regression further identified an inflection point at approximately 92 minutes per day (95% CI 75‐113 min/d), below which symptom severity increased sharply and above which scores plateaued. Likelihood ratio testing confirmed the segmented nonlinear pattern (*P*<.001), reinforcing the graded dose-response relationship implied by the 70 minutes per day risk threshold observed in our main logistic models. Full piecewise linear regression outputs are summarized in Table S9 in Multimedia Appendix 1.

### Multivariable Logistic Regression Analysis of Screen Time and Mental Health Outcomes

The associations between daily screen time and 3 mental health outcomes are presented in Table 2 (the unadjusted model results are in Table S10 in Multimedia Appendix 1). In adjusted models, each 1-hour increase in average daily screen time was associated with a 19% increase in the odds of total difficulties (OR 1.19, 95% CI 1.15‐1.23), and with 15% and 12% increases in externalizing problems (OR 1.15, 95% CI 1.11‐1.18) and internalizing problems (OR 1.12, 95% CI 1.09‐1.16), respectively.

Using weekday screen-time categories (reference: <30 min/d), a clear dose-response pattern emerged: the adjusted ORs for total difficulties increased from 1.31 (30‐59 min) to 1.75 (60‐119 min), 1.89 (120‐180 min), and 1.76 (>180 min), with similar gradients for externalizing and internalizing problems. In contrast, weekend screen time showed weaker associations; only the high categories (≥120 min) reached statistical significance. These results indicate that weekday screen time is more strongly and consistently associated with children’s mental health than weekend screen time.

Unadjusted crude effect estimates are presented in Table S10 in Multimedia Appendix 1.

**Table 2.** Multivariable logistic regression between screen time and mental health outcomes (N=21,366)^a^.

Exposure	Total difficulties, OR^b^ (95% CI)	Externalizing problems, OR (95% CI)	Internalizing problems, OR (95% CI)
Average daily screen time			
Per 1-hour increase	1.19 (1.15‐1.23)	1.15 (1.11‐1.18)	1.12 (1.09‐1.16)
Daily screen time (weekdays, min)			
<30	1.0 (reference)	1.0 (reference)	1.0 (reference)
30-59	1.31 (1.20‐1.43)	1.24 (1.15‐1.34)	1.16 (1.07‐1.26)
60-119	1.75 (1.56‐1.95)	1.44 (1.31‐1.60)	1.54 (1.38‐1.71)
120-180	1.89 (1.60‐2.23)	1.64 (1.40‐1.91)	1.55 (1.32‐1.82)
＞180	1.76 (1.57‐1.98)	1.55 (1.39‐1.72)	1.40 (1.25‐1.56)
Daily screen time (weekends, min)			
<30	1.0 (reference)	1.0 (reference)	1.0 (reference)
30-59	1.01 (0.91‐1.11)	1.03 (0.94‐1.12)	0.94 (0.86‐1.04)
60-119	1.09 (0.98‐1.22)	1.16 (1.05‐1.28)	1.04 (0.94‐1.16)
120-180	1.34 (1.17‐1.53)	1.28 (1.13‐1.45)	1.21 (1.06‐1.38)
>180	1.45 (1.30‐1.62)	1.33 (1.20‐1.47)	1.23 (1.11‐1.37)

a Adjusted for child gender, child age, primary parent gender, primary parent age, smoking and alcohol use, number of children, marital status, education level, occupation, family income, and children’s household registration.

b OR: odds ratio.

### Subgroup and Interaction Analyses

**Figure 2.**
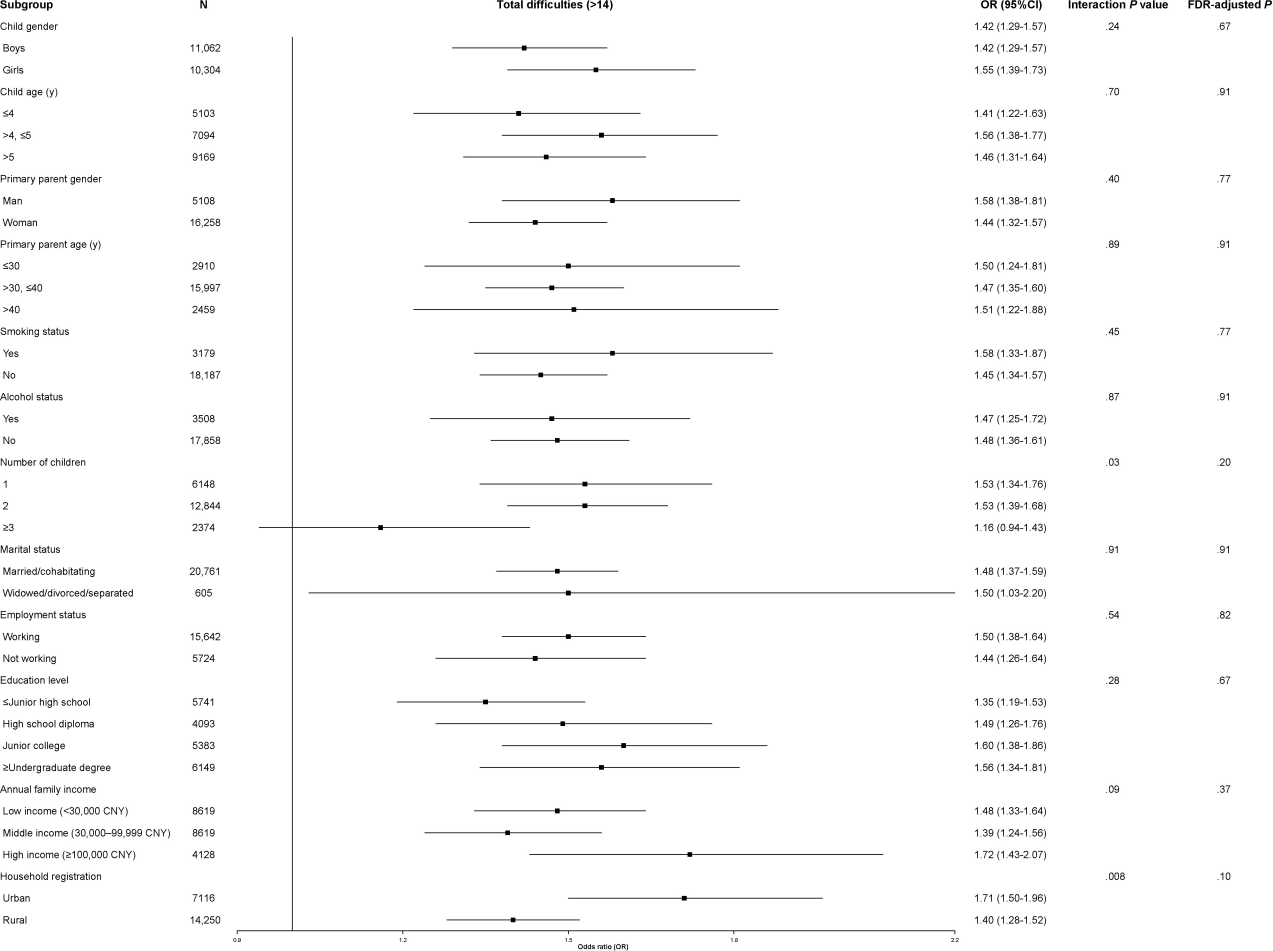
Subgroup analysis of the association between average daily screen time and mental health problems (total difficulties score [TDS] >14) across various subgroups. Stratification was implemented based on the originally estimated break point of 70.71 minutes per day from 2-piece logistic regression. Average daily screen time ≤70.71 minutes per day was used as the reference. Analyses were adjusted for all covariates except the stratifier. FDR: false discovery rate.

Subgroup analyses were performed to examine whether the association between high screen time (threshold ≥ approximately 70 min/d) and total difficulties varied across sociodemographic characteristics (Figure 2). Potential interactions were suggested for the number of children (raw *P*=.03) and household registration (raw *P*=.008), although neither interaction remained statistically significant after FDR correction for multiple comparisons (adjusted *P*=.20 and .10, respectively). No other significant interactions were detected.

### Indirect Effect Through Parental Discipline

Sequential regression models demonstrated a gradual attenuation of ORs for elevated screen exposure (above approximately 70 min/d) after adjustment for parenting practices alone and further after adding all predefined covariates (Table 3). This pattern of attenuation suggests that parental discipline serves as an intermediate correlational factor in the associations between excessive screen time and children’s mental health outcomes, as observed in this cross-sectional dataset.

**Figure 3.**
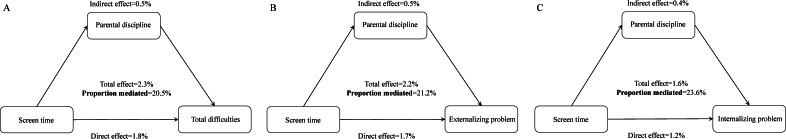
The indirect effect analysis of screen time on children’s mental health outcomes through parental discipline (N=21,366). (A) Total difficulties, (B) externalizing problems, and (C) internalizing problems.

The average proportion mediated by parental discipline was 20.5% for total difficulties (95% CI 15.8%‐26.2%), 21.2% for externalizing problems (95% CI 15.7%‐28.4%), and 23.6% for internalizing problems (95% CI 17.2%‐33.5%), respectively (Table 4 and Figure 3). Parental discipline accounted for approximately one-fifth of the observed association between average daily screen time and children’s mental health outcomes. Given the observed indirect effect estimates and a sample size of 21,366, post hoc power analysis suggested sufficient statistical power (>.80) to detect the mediating effects in this cross-sectional study [53].

Subscale-resolved mediation analysis showed that overreactivity accounted for the largest proportion of the indirect effect across all outcomes (20.1% for total difficulties, 23.0% for externalizing problems, and 23.0% for internalizing problems), whereas laxness showed only minimal mediation (2.7%‐4.0%), and verbosity did not show meaningful indirect effects (the proportion mediated was negative or near zero). These patterns are fully documented in Table S11 in Multimedia Appendix 1.

**Table 3.** Changes in the association between high screen time and children’s mental health outcomes with sequential adjustment for parental discipline (PS) (N=21,366)^a^.

Outcome	Crude, OR^b^ (95% CI)	Model 1 (adjusted PS only), OR (95% CI)	Model 2 (adjusted all covariates plus PS), OR (95% CI)
Total difficulties	1.53 (1.42‐1.64)	1.44 (1.34‐1.55)	1.40 (1.30‐1.51)
Externalizing problems	1.31 (1.22‐1.39)	1.24 (1.16‐1.32)	1.24 (1.16‐1.33)
Internalizing problems	1.37 (1.27‐1.46)	1.29 (1.21‐1.39)	1.27 (1.18‐1.36)

a Stratification for high screen time was defined using the precise estimated threshold of 70.71 minutes per day derived from piecewise logistic regression. Crude model: adjusted for none; model 1: adjusted for PS only; model 2: adjusted for PS, child gender, child age, primary parent gender, primary parent age, smoking and alcohol use, number of children, marital status, education level, occupation, family income, and children’s household registration.

b OR: odds ratio.

**Table 4.** The indirect effect analysis of screen time on children’s mental health outcomes through parental discipline (N=21,366)^a^.

Effect type	Total difficulties, estimate (95% CI)	Externalizing problems, estimate (95% CI)	Internalizing problems, estimate (95% CI)
Total effect	0.023 (0.019‐0.027)	0.022 (0.012‐0.021)	0.016 (0.012‐0.021)
Mediation effect (average)	0.005 (0.004‐0.006)	0.005 (0.004‐0.006)	0.004 (0.003‐0.005)
Direct effect (average)	0.018 (0.014‐0.022)	0.017 (0.012‐0.022)	0.012 (0.008‐0.017)
Proportion mediated (average)	0.205 (0.158‐0.262)	0.212 (0.157‐0.284)	0.236 (0.172‐0.335)

a Adjusted for child gender, child age, primary parent gender, primary parent age, smoking and alcohol use, number of children, marital status, education level, occupation, family income, and children’s household registration.

### Sensitivity Analyses

Several sensitivity analyses were performed to test the robustness of the main findings. The results from the threshold-based analysis using screen time dichotomized at 70.71 minutes were consistent with the primary results (Table S12 in Multimedia Appendix 1). After excluding participants with extreme weekday screen time (≥180 min), the results remained essentially unchanged (Table S13 in Multimedia Appendix 1). Furthermore, E-values ranged from 1.55 to 1.73, indicating moderate robustness against potential unmeasured confounding (Table S14 in Multimedia Appendix 1). The multiple imputation analysis also yielded estimates consistent with the complete-case analysis, with similar dose-response patterns and significance levels (Table S15 in Multimedia Appendix 1). In the reverse-direction sensitivity analysis, the proportion mediated was consistently lower than in the forward model for all 3 outcomes: 13.9% vs 20.5% for total difficulties, 16.6% vs 21.2% for externalizing, and 19.2% vs 23.6% for internalizing problems (Table S16 in Multimedia Appendix 1). Although the mediated share was larger under our hypothesized directional structure, all reverse indirect associations remained statistically significant, and cross-sectional data cannot resolve the true temporal ordering of these variables. Collectively, these sensitivity analyses support the stability of the main conclusions.

## Discussion

### Principal Findings

In this cross-sectional study of 21,366 parent-child dyads from Western China, we observed a nonlinear association between average daily screen time and children’s mental health outcomes. The risk rose steeply at lower exposure levels and gradually plateaued at higher levels, with a model-derived threshold of approximately 70 minutes per day (95% CI 66-75 min). Weekday screen time exhibited a stronger and more consistent dose-response relationship with children’s mental health than weekend screen time. Subgroup analyses suggested potential moderation by the number of children and household registration, although these interactions did not remain statistically significant after FDR correction for multiple comparisons. Finally, parental discipline accounted for approximately 20.5% to 23.6% of the association between screen time and mental health outcomes, suggesting a potential indirect statistical association.

### Nonlinear Relationship and Threshold Effect

A nonlinear relationship was observed between screen exposure and mental health outcomes in preschool children, with a saturating dose-response pattern. A 2-piecewise logistic regression reparameterization yielded a policy-interpretable summary of approximately 70 minutes per day (95% CI 66‐75) for elevated clinical risk. We assessed model performance via AIC comparison; the GAM exhibited marginally better statistical fit (ΔAIC=−6.78), indicating that the dose-response relationship is better characterized as a graded, saturating curve than a sharply defined break point. Nonetheless, the absolute difference between models was modest (|ΔAIC|<10). We retained the 2-piecewise framework for the primary results because it provides a policy-interpretable summary—the approximately 70 minutes per day value—that facilitates translation for clinical and public health audiences, while acknowledging that this value is a reparameterization of the GAM-derived curve rather than a data-selected “true” threshold. Below this summary estimate, risk increased rapidly with longer screen use, whereas the marginal increase in risk diminished at higher exposure levels, consistent with evidence from population-based studies on early digital media use [34,54,55]. Notably, the association for internalizing problems showed a more complex, fluctuating pattern (estimated *df*=5.62), which may be related to screen content types or individual differences in susceptibility. These patterns may reflect several mechanisms. First, the displacement of protective activities (eg, physical activity and parent-child interaction) may occur even at low screen doses, whereas very high exposure often coincides with other unmeasured family stressors that dominate the association and create a ceiling effect [56-58]. Second, sleep disruption is a well-documented mediator that may saturate after a certain screen level, as additional hours no longer further reduce sleep duration in children already experiencing poor sleep hygiene [59-61]. Third, high-dose exposure may induce tolerance or compensatory adaptation in neural responses [62].

From a digital health perspective, this policy-interpretable summary of approximately 70 minutes per day (95% CI 66‐75), while dependent on the analytical specification of the 2-piecewise regression, provides a quantifiable target for family-based interventions and shows numerical convergence with existing pediatric guidelines (eg, <1  h/d for preschoolers) [63]. This finding may offer a useful reference point that reflects the evolving reality of increasing digital device use in contemporary society. Although several studies have already used regression models to examine children’s screen time [39,64,65], our work extends this research specifically for the mental health of preschool children by identifying data-driven thresholds rather than arbitrary limits, which offers local contextual reference for digital parenting efforts in Western China.

Notably, the inflection point derived from continuous total difficulty scores (92 min/d) diverges from the 70 minutes per day threshold identified in binary logistic models. This difference aligns with methodological logic. The 70-minute threshold demarcates screen-time exposure associated with elevated clinical behavioral risk, whereas the 92-minute break point captures the time point at which the growth rate of symptom severity plateaus. Collectively, these complementary thresholds demonstrate a graded dose-response pattern between screen time and children’s mental health, addressing the analytical limitation of relying solely on a single binary SDQ cutoff. The parent-reported SDQ shows acceptable cross-cultural measurement invariance for Chinese preschoolers and is appropriate for this analysis. However, divergent parental symptom reporting styles across cultural contexts restrict broad generalization to other ethnic or national cohorts.

### Weekday vs Weekend Screen Time: Contextual Patterns and Personalized Digital Health

Our findings showed that weekday screen time exhibited a stronger and more consistent dose-response association with children’s mental health than weekend screen time. This pattern difference may reflect that the context and behavioral patterns of digital media use may be more influential than the total daily duration alone [66,67]. Weekday screen exposure likely reflects more structured, education-related, or supervised use, with greater regularity and lower within-family variability, which may strengthen its independent association with children’s mental health. In contrast, weekend screen time tends to involve more recreational content, unstructured passive viewing, and shared family media use, which may be accompanied by shared viewing or parental supervision, potentially mitigating adverse effects [68-70]. It is important to acknowledge that our data captured only screen-time duration and did not distinguish content type, device, or coviewing context. The observed weekday/weekend differences may reflect variations in the nature and context of screen use, but this interpretation remains speculative and requires confirmation in future studies with detailed content and context measures.

These differential patterns underscore the importance of timing, setting, and content type in interpreting screen-related risks [71]. From a digital health perspective, interventions should prioritize weekday screen-time management as a more impactful and modifiable target, while allowing reasonable flexibility on weekends. Such context-specific recommendations align with contemporary digital health frameworks, emphasizing personalized, scenario-based guidance rather than one-size-fits-all restrictions [72]. These findings further support the value of stratifying screen time by context to improve the precision of family-focused digital health strategies.

### Digital Health Disparities: Exploratory Subgroup Trends by Family Size and Household Registration

Subgroup analyses suggested potential effect modification by family size and household registration, although these interactions did not remain statistically significant after FDR correction for multiple comparisons. The observed patterns—weaker associations in multichild families and stronger associations in urban households—should only be viewed as preliminary exploratory trends rather than confirmed robust findings. In multichild families, sibling co-viewing, shared device use, and peer buffering may reduce the harmful impact of screen exposure, as children interact with each other during screen time rather than engaging in solitary, unregulated consumption [73,74]. The stronger effect observed among urban children may reflect higher device accessibility, greater prevalence of solitary smartphone/tablet use, and fewer alternative outdoor activities compared with rural settings [75,76].

Notably, the seemingly weaker associations in rural children do not imply lower risk but may reflect distinct digital-divide profiles, including lower device quality, limited high-quality content, and uneven access to beneficial digital resources [77,78]. This tentative pattern is shaped by China’s household registration (*hukou*) system—the birth-based urban-vs-rural classification that determines access to locally funded public services such as education and health care [79]. Such institutional barriers may constrain the extent to which increased family income can be translated into improved digital resources and health services for rural families [80]. In Western China, digital-device penetration is dominated by televisions, tablets, and mobile phones, while access to desktop computers and interactive educational media remains less prevalent compared with more developed urban regions. Given the lack of statistical significance after multiplicity adjustment, these exploratory subgroup observations cannot reliably support targeted equity-centered digital health interventions at present. Additional large-scale confirmatory research is needed to verify these subgroup differences before differentiated parenting guidelines can be prioritized.

### Indirect Effect: The Mediating Role of Parental Discipline

Our mediation analysis indicated that parental discipline accounted for approximately 20% to 24% of the association between screen time and children’s mental health problems, reflecting statistical mediation under cross-sectional assumptions rather than a causal mechanism. Subscale-resolved mediation further revealed that this indirect association was primarily driven by the over-reactivity dimension; laxness yielded trivial positive mediating effects, whereas verbosity showed weak negative indirect contributions. Prolonged screen exposure may reduce the quality of parent-child interaction, increase conflicts over device use, and demand constant monitoring of content and duration, thereby elevating parental stress and harsh or inconsistent disciplinary responses [81,82]. In turn, disrupted parenting practices further exacerbate emotional and behavioral difficulties in young children [23]. Notably, this pathway may be bidirectional in daily family dynamics: parents experiencing greater discipline stress may also rely more heavily on screen devices as a calming or childcare strategy, creating a mutually reinforcing cycle.

These findings support the view from previous studies that parents act as critical gatekeepers in children’s digital media exposure, particularly during early childhood [83]. From a digital health perspective, family-based interventions targeting improved parent-child communication, alternative engagement activities, and parental self-efficacy may help buffer the adverse effects of excessive screen use [30,84,85]. Because this is a cross-sectional design, no causal inference can be drawn. The reverse sensitivity analysis showed a smaller proportion of the total effect mediated by parental discipline (13.9%‐19.2%) than the forward model (20.5%‐23.6%). While the forward correlational linkages exhibited larger relative mediated contributions, all reverse models still yielded statistically significant indirect associations, confirming that bidirectional correlational patterns fit the observed data. All results reported herein should be interpreted only as correlational associations rather than as causal sequential pathways. Culturally rooted Chinese parenting norms centered on academic achievement and child behavioral compliance may jointly shape parents’ disciplinary approaches and daily screen management practices for preschoolers.

### Limitations and Future Directions

Several limitations should be acknowledged. First, the cross-sectional design precludes causal inference, and findings should be interpreted as associational rather than directional, even with statistically significant indirect effects observed in the mediation analysis. Second, all measures relied on parent-reported data, and teacher-rated SDQ data were not collected. Future studies integrating both parent and teacher evaluations could reduce single-source bias and provide multi-informant validation. Third, we captured only screen duration and lacked information about content quality, educational vs entertainment media, co-viewing, or interactive versus passive engagement, limiting our ability to distinguish differential effects across content types or contexts. Fourth, the data were collected from a single western region of China. Our sample from Yan’an (Shaanxi) represents midtier non–provincial-capital western cities in terms of per capita gross domestic product and urban-rural distribution, and stratified cluster sampling across all 13 districts supports regional representativeness of local 3- to 6-year-old community preschoolers. However, the results cannot be generalized to other age groups, populations with divergent socioeconomic or cultural environments, or clinical samples with pre-existing child psychiatric disorders. Fifth, data collection occurred nearly 1 month after the 2025 Spring Festival holiday. Local parents and preschools had resumed normal routines, minimizing holiday-specific atypical screen use and childcare patterns, though subtle residual seasonal effects cannot be fully ruled out. Finally, E-values ranged from 1.55 to 1.73, indicating moderate robustness against unmeasured confounding. Given the 18.6% prevalence of elevated SDQ difficulties, an E-value below 2.0 means a moderately strong omitted confounder (parental mental health, household chaos, child sleep duration, pre-existing child behavioral vulnerabilities, screen content type, and parent-child interaction quality) could substantially attenuate our association. These factors likely overestimate the screen time–symptom association, as children with poorer baseline regulation or parents with depressive symptoms may rely more heavily on screens for childcare. However, only a confounder exceeding our E-value thresholds could fully erase the graded dose-response trend; weaker residual bias cannot reverse the positive association. Future longitudinal studies with objective screen-use measures (eg, digital logs and wearable sensors) and randomized controlled trials of family-centered digital parenting interventions are needed to strengthen causal inference.

### Conclusions

This cross-sectional study identifies a nonlinear association between screen time and preschool mental health outcomes, with a model-derived threshold identified at approximately 70 minutes per day, and offers preliminary correlational evidence that parental discipline may be statistically associated as an intermediate factor in this relationship. These findings offer a preliminary local reference for region-specific screen-time management and highlight potentially modifiable parenting factors, with implications for digital health interventions focused on early childhood mental health.

## Supplementary material

10.2196/99762 Multimedia Appendix 1 Supplementary tables and the figure containing variance inflation factor results, participant baseline characteristics, correlation matrices, generalized additive model outputs, threshold analyses, logistic regression, mediation models, and multiple sensitivity analyses.

10.2196/99762 Checklist 1 STROBE checklist.

## Abbreviations

AIC: Akaike information criterion
FDR: false discovery rate
GAM: generalized additive model
OR: odds ratio
SDQ: Strengths and Difficulties Questionnaire
STROBE: Strengthening the Reporting of Observational Studies in Epidemiology
