# Supplementary material for: Digital Screen Time and Mental Health Difficulties Among Preschool Children in Western China: Cross-Sectional Study
Source: J Med Internet Res. 2026 Aug 17;28:e99762. doi: 10.2196/99762 (PMC13480941; doi:10.2196/99762)
Supplement: Supplementary file 1 [file jmir-v28-e99762-s002.pdf]

STROBE Statement—Checklist of items that should be included in reports of ***cross-sectional studies***

	Item No	Recommendation	Reported on manuscript page
**Title and abstract**	1	(*a*) Indicate the study's design with a commonly used term in the title or the abstract	Page1-2 (Abstract)
		(*b*) Provide in the abstract an informative and balanced summary of what was done and what was found	Page1-2 (Abstract)
**Introduction**			
Background/rationale	2	Explain the scientific background and rationale for the investigation being reported	Page2-4(Introduction）
Objectives	3	State specific objectives, including any prespecified hypotheses	Page4(Introduction: last paragraph）
**Methods**			
Study design	4	Present key elements of study design early in the paper	Page4-5(Methods:Study design and sample)
Setting	5	Describe the setting, locations, and relevant dates, including periods of recruitment, exposure, follow-up, and data collection	Page4-5(Methods:Study design and sample)
Participants	6	(*a*) Give the eligibility criteria, and the sources and methods of selection of participants	Page4-5(Methods:Study design and sample)
Variables	7	Clearly define all outcomes, exposures, predictors, potential confounders, and effect modifiers. Give diagnostic criteria, if applicable	Page5-6(Methods: Measures)
Data sources/ measurement	8*	For each variable of interest, give sources of data and details of methods of assessment (measurement). Describe comparability of assessment methods if there is more than one group	Page5-6(Methods: Measures)
Bias	9	Describe any efforts to address potential sources of bias	Page6-8(Methods:Statistical analysis); 19(Limitations)
Study size	10	Explain how the study size was arrived at	Page4-5(Methods:Study design and sample)
Quantitative variables	11	Explain how quantitative variables were handled in the analyses. If applicable, describe which groupings were chosen and why	Page5-6(Methods: Measures)
Statistical methods	12	(*a*) Describe all statistical methods, including those used to control for confounding	Page6-8 (Methods:Statistical analysis)
		(*b*) Describe any methods used to examine subgroups and interactions	Page7 (Methods:Statistical analysis)
		(*c*) Explain how missing data were addressed	Page7 (Methods:Statistical analysis)
		(*d*) If applicable, describe analytical methods taking account of sampling strategy	Page4-5(Methods:Study design and sample)
		(*e*) Describe any sensitivity analyses	Page7 (Methods:Statistical analysis); Page15(Results: Sensitivity analyses, TableS6-8)
**Results**			

Participants	13*	(a) Report numbers of individuals at each stage of study—eg numbers potentially eligible, examined for eligibility, confirmed eligible, included in the study, completing follow-up, and analysed	Page4-5(Methods:Study design and sample)
		(b) Give reasons for non-participation at each stage	Page4-5(Methods:Study design and sample)
		(c) Consider use of a flow diagram	
Descriptive data	14*	(a) Give characteristics of study participants (eg demographic, clinical, social) and information on exposures and potential confounders	Page8-10 (Results:3.1 Characteristics of the study population, Table1; Table S2)
		(b) Indicate number of participants with missing data for each variable of interest	Page4-5(Methods:Study design and sample)
Outcome data	15*	Report numbers of outcome events or summary measures	Page8-15 (Results:3.1-3.5)
Main results	16	(*a*) Give unadjusted estimates and, if applicable, confounder-adjusted estimates and their precision (eg, 95% confidence interval). Make clear which confounders were adjusted for and why they were included	Page8-15 (Results:3.1-3.5;Tables 1-4)
		(*b*) Report category boundaries when continuous variables were categorized	Page5-6 (Methods: Measures)
		(*c*) If relevant, consider translating estimates of relative risk into absolute risk for a meaningful time period	Not applicable
Other analyses	17	Report other analyses done—eg analyses of subgroups and interactions, and sensitivity analyses	Page9-10 (Results:3.5-3.6;Fig 2, TableS6-8)
**Discussion**			
Key results	18	Summarise key results with reference to study objectives	Page15-16(Discussion)
Limitations	19	Discuss limitations of the study, taking into account sources of potential bias or imprecision. Discuss both direction and magnitude of any potential bias	Page19(Limitations)
Interpretation	20	Give a cautious overall interpretation of results considering objectives, limitations, multiplicity of analyses, results from similar studies, and other relevant evidence	Page15-19(Discussion)
Generalisability	21	Discuss the generalisability (external validity) of the study results	Page19(Limitations)
**Other information**			
Funding	22	Give the source of funding and the role of the funders for the present study and, if applicable, for the original study on which the present article is based	Not applicable

*Give information separately for exposed and unexposed groups.
