# Supplementary material for: Digital Screen Time and Mental Health Difficulties Among Preschool Children in Western China: Cross-Sectional Study
Source: J Med Internet Res. 2026 Aug 17;28:e99762. doi: 10.2196/99762 (PMC13480941; doi:10.2196/99762)
Supplement: Supplementary file 2 [file jmir-v28-e99762-s001.docx]

Table S1 the VIF values of independent variables and covariates

| **Variable** | **VIF** |
| --- | --- |
| Total difficulties | 2.3 |
| Externalizing problem | 1.5 |
| Internalizing problem | 1.8 |
| Average daily screen time | 1.0 |
| Parental discipline (PS) | 1.1 |
| Child gender | 1.0 |
| Child age, years | 1.0 |
| Primary parent gender | 2.4 |
| Primary parent age, years | 1.2 |
| Smoking status | 2.3 |
| Alcohol status | 1.8 |
| Number of children | 1.2 |
| Marital status | 1.0 |
| Education level | 1.6 |
| Occupation | 1.4 |
| Annual family income | 1.2 |
| Household registration of children | 1.3 |

VIF = variance inflation factor, calculated as 1/(1-R²). Stepwise screening was applied with a threshold of 5. All variables exhibited VIF < 5, indicating no substantial multicollinearity.

Table S2 Characteristics of participants by externalizing and internalizing problems in children (N=21,366)

| **Variable** | **Externalizing problems** | | ***P*-value** | **Internalizing problems** | | ***P*-value** |
| --- | --- | --- | --- | --- | --- | --- |
|  | **Normal**  **(**N**=**16205, 75.8%**)** | **At risk of mental health problems**  **(**N**=**5161, 24.2%**)** |  | **Normal**  **(**N=16906, 79.1%**)** | **At risk of mental health problems**  **(**N=4460, 20.9%**)** |  |
|  | Mean (SD) | | | | |  |
| **Child age, years** | 4.83 (0.88) | 4.77 (0.90) | <.001 | 4.81 (0.89) | 4.85 (0.88) | .005 |
| **Primary parent age, years** | 34.80 (4.56) | 34.59 (4.56) | <.001 | 34.81 (4.56) | 34.53 (4.57) | <.001 |
| **Parental discipline (PS)** | 111.26 (13.70) | 116.72 (10.93) | <.001 | 111.47 (13.62) | 116.80 (10.97) | <.001 |
| **Average daily screen time (min)** | 65.26 (57.26) | 73.51 (61.42) | <.001 | 65.56 (57.44) | 73.67 (61.47) | <.001 |
|  | N (%) | | | | |  |
| **Child gender** |  |  | <.001 |  |  | .014 |
| Boys | 8268 (51.0%) | 2794 (54.1%) |  | 8826 (52.2%) | 2236 (50.1%) |  |
| Girls | 7937 (49.0%) | 2367 (45.9%) |  | 8080 (47.8%) | 2224 (49.9%) |  |
| **Primary parent gender** |  |  | <.001 |  |  | <.001 |
| Male | 3779 (23.3%) | 1329 (25.8%) |  | 3865 (22.9%) | 1243 (27.9%) |  |
| Female | 12426 (76.7%) | 3832 (74.3%) |  | 13041 (77.1%) | 3217 (72.1%) |  |
| **Smoking history** |  |  | .003 |  |  | <.001 |
| Yes | 2346 (14.5%) | 833 (16.1%) |  | 2378 (14.1%) | 801 (18.0%) |  |
| No | 13859 (85.5%) | 4328 (83.9%) |  | 14528 (85.9%) | 3659 (82.0%) |  |
| **Alcohol history** |  |  | <.001 |  |  | <.001 |
| Yes | 2554 (15.8%) | 954 (18.5%) |  | 2638 (15.6%) | 870 (19.5%) |  |
| No | 13651 (84.2%) | 4207 (81.5%) |  | 14268 (84.4%) | 3590 (80.5%) |  |
| **Number of children** |  |  | .031 |  |  | <.001 |
| 1 | 4637 (28.6%) | 1511 (29.3%) |  | 4879 (28.9%) | 1269 (28.5%) |  |
| 2 | 9716 (60.0%) | 3128 (60.6%) |  | 10232 (60.5%) | 2612 (58.6%) |  |
| ≥ 3 | 1852 (11.4%) | 522 (10.1%) |  | 1795 (10.6%) | 579 (13.0%) |  |
| **Marital status** |  |  | .182 |  |  | <.001 |
| Married/cohabitating | 15760 (97.3%) | 5001 (96.9%) |  | 16473 (97.4%) | 4288 (96.1%) |  |
| Widowed/divorced/separated | 445 (2.7%) | 160 (3.1%) |  | 433 (2.6%) | 172 (3.9%) |  |
| **Employment status** |  |  | .420 |  |  | <.001 |
| Working | 11886 (73.4%) | 3756 (72.8%) |  | 12617 (74.6%) | 3025 (67.8%) |  |
| Not working | 4319 (26.6%) | 1405 (27.2%) |  | 4289 (25.4%) | 1435 (32.2%) |  |
| **Education level** |  |  | .014 |  |  | <.001 |
| ≤ Junior high school | 4273 (26.4%) | 1468 (28.4%) |  | 4224 (25.0%) | 1517 (34.0%) |  |
| High school diploma | 3157 (19.5%) | 936 (18.1%) |  | 3236 (19.1%) | 857 (19.2%) |  |
| junior college | 4109 (25.4%) | 1274 (24.7%) |  | 4327 (25.6%) | 1056 (23.7%) |  |
| ≥ Undergraduate degree | 4666 (28.8%) | 1483 (28.7%) |  | 5119 (30.3%) | 1030 (23.1%) |  |
| **Annual family income, CNY** |  |  | <.001 |  |  | <.001 |
| <30,000 | 6249 (38.6%) | 2370 (45.9%) |  | 6459 (38.2%) | 2160 (48.4%) |  |
| 30,000 to 99,999 | 6725 (41.5%) | 1894 (36.7%) |  | 6960 (41.2%) | 1659 (37.2%) |  |
| ≥100,000 | 3231 (19.9%) | 897 (17.4%) |  | 3487 (20.6%) | 641 (14.4%) |  |
| **Household registration of children** |  |  | .458 |  |  | <.001 |
| Urban | 5419 (33.4%) | 1697 (32.9%) |  | 5830 (34.5%) | 1286 (28.8%) |  |
| rural | 10786 (66.6%) | 3464 (67.1%) |  | 11076 (65.5%) | 3174 (71.2%) |  |
| **Daily screen time (min)** |  |  |  |  |  |  |
| **weekdays** |  |  | <.001 |  |  | <.001 |
| <30 min | 5770 (35.6%) | 1502 (29.1%) |  | 5954 (35.2%) | 1318 (29.6%) |  |
| 30 to 59 min | 6033 (37.2%) | 1927 (37.3%) |  | 6361 (37.6%) | 1599 (35.9%) |  |
| 60 to 119 min | 2049 (12.6%) | 764 (14.8%) |  | 2105 (12.5%) | 708 (15.9%) |  |
| 120 to 179 min | 638 (3.9%) | 270 (5.2%) |  | 673 (4.0%) | 235 (5.3%) |  |
| ≥180 min | 1715 (10.6%) | 698 (13.5%) |  | 1813 (10.7%) | 600 (13.5%) |  |
| **weekends** |  |  | <.001 |  |  | <.001 |
| <30 min | 3463 (21.4%) | 1016 (19.7%) |  | 3548 (21.0%) | 931 (20.9%) |  |
| 30 to 59 min | 5498 (33.9%) | 1601 (31.0%) |  | 5753 (34.0%) | 1346 (30.2%) |  |
| 60 to 119 min | 3352 (20.7%) | 1090 (21.1%) |  | 3543 (21.0%) | 899 (20.2%) |  |
| 120 to 179 min | 1426 (8.8%) | 511 (9.9%) |  | 1491 (8.8%) | 446 (10.0%) |  |
| ≥180 min | 2466 (15.2%) | 943 (18.3%) |  | 2571 (15.2%) | 838 (18.8%) |  |

Note: Continuous variables are expressed as mean±SD. Categorical variables are expressed as frequency (%).

Table S3 Baseline characteristics of the study participants by weekday daily screen time (N=21,366)

| **Variable** |  | **Weekday daily screen time (min)** | | | | | ***P*-value** |
| --- | --- | --- | --- | --- | --- | --- | --- |
|  |  | <30 min | 30 to 59 min | 60 to 119 min | 120 to 179 min | ≥180 min |  |
|  |  | **N=**7272 | **N=**7960 | **N=**2813 | **N=**908 | **N=**2413 |  |
|  |  | Mean (SD) | | | | |  |
| **Child age, years** |  | 4.80 (0.90) | 4.82 (0.88) | 4.82 (0.87) | 4.86 (0.88) | 4.82 (0.90) | .225 |
| **Primary parent age, years** |  | 34.62 (4.54) | 34.74 (4.54) | 35.21 (4.67) | 35.32 (4.44) | 34.42 (4.60) | <.001 |
| **Parental discipline (PS)** |  | 111.24 (13.96) | 112.38 (13.23) | 114.65 (12.27) | 114.55 (11.81) | 114.13 (12.54) | <.001 |
|  |  | N (%) | | | | |  |
| **Child gender** |  |  |  |  |  |  | .875 |
| Boys |  | 3564 (49.0%) | 4220 (53.0%) | 1506 (53.5%) | 513 (56.5%) | 1259 (52.2%) |  |
| Girls |  | 3708 (51.0%) | 3740 (47.0%) | 1307 (46.5%) | 395 (43.5%) | 1154 (47.8%) |  |
| **Primary parent gender** |  |  |  |  |  |  | <.001 |
| Male |  | 1648 (22.7%) | 1850 (23.2%) | 751 (26.7%) | 268 (29.5%) | 591 (24.5%) |  |
| Female |  | 5624 (77.3%) | 6110 (76.8%) | 2062 (73.3%) | 640 (70.5%) | 1822 (75.5%) |  |
| **Smoking status** |  |  |  |  |  |  | <.001 |
| Yes |  | 959 (13.2%) | 1168 (14.7%) | 473 (16.8%) | 163 (18.0%) | 416 (17.2%) |  |
| No |  | 6313 (86.8%) | 6792 (85.3%) | 2340 (83.2%) | 745 (82.0%) | 1997 (82.8%) |  |
| **Alcohol status** |  |  |  |  |  |  | <.001 |
| Yes |  | 968 (13.3%) | 1290 (16.2%) | 574 (20.4%) | 220 (24.2%) | 456 (18.9%) |  |
| No |  | 6304 (86.7%) | 6670 (83.8%) | 2239 (79.6%) | 688 (75.8%) | 1957 (81.1%) |  |
| **Number of children** |  |  |  |  |  |  | <.001 |
| 1 |  | 2083 (28.6%) | 2404 (30.2%) | 767 (27.3%) | 222 (24.5%) | 672 (27.9%) |  |
| 2 |  | 4421 (60.8%) | 4744 (59.6%) | 1701 (60.5%) | 545 (60.0%) | 1433 (59.4%) |  |
| ≥ 3 |  | 768 (10.6%) | 812 (10.2%) | 345 (12.3%) | 141 (15.5%) | 308 (12.8%) |  |
| **Marital status** |  |  |  |  |  |  | .005 |
| Married/cohabitating |  | 7058 (97.1%) | 7762 (97.5%) | 2744 (97.6%) | 875 (96.4%) | 2322 (96.2%) |  |
| Widowed/divorced/separated |  | 214 (2.9%) | 198 (2.5%) | 69 (2.4%) | 33 (3.6%) | 91 (3.8%) |  |
| **Employment status** |  |  |  |  |  |  | <.001 |
| Working |  | 5337 (73.4%) | 5963 (74.9%) | 2057 (73.1%) | 664 (73.1%) | 1621 (67.2%) |  |
| Not working |  | 1935 (26.6%) | 1997 (25.1%) | 756 (26.9%) | 244 (26.9%) | 792 (32.8%) |  |
| **Education level** |  |  |  |  |  |  | <.001 |
| ≤ Junior high school |  | 1894 (26.1%) | 1923 (24.2%) | 760 (27.0%) | 288 (31.7%) | 876 (36.3%) |  |
| High school diploma |  | 1391 (19.1%) | 1528 (19.2%) | 508 (18.1%) | 159 (17.5%) | 507 (21.0%) |  |
| junior college |  | 1820 (25.0%) | 2068 (26.0%) | 720 (25.6%) | 229 (25.2%) | 546 (22.6%) |  |
| ≥ Undergraduate degree |  | 2167 (29.8%) | 2441 (30.7%) | 825 (29.3%) | 232 (25.6%) | 484 (20.1%) |  |
| **Annual family income** |  |  |  |  |  |  | <.001 |
| ≤ 100 000 CNY |  | 3079 (42.3%) | 3040 (38.2%) | 1071 (38.1%) | 350 (38.6%) | 1079 (44.7%) |  |
| >100 000 to 300 000 CNY |  | 2788 (38.3%) | 3360 (42.2%) | 1166 (41.5%) | 378 (41.6%) | 927 (38.4%) |  |
| > 300 000 CNY |  | 1405 (19.3%) | 1560 (19.6%) | 576 (20.5%) | 180 (19.8%) | 407 (16.9%) |  |
| **Household registration of children** |  |  |  |  |  |  | <.001 |
| Urban |  | 2483 (34.1%) | 2735 (34.4%) | 962 (34.2%) | 293 (32.3%) | 643 (26.7%) |  |
| rural |  | 4789 (65.9%) | 5225 (65.6%) | 1851 (65.8%) | 615 (67.7%) | 1770 (73.3%) |  |

Note: Continuous variables are expressed as mean±SD. Categorical variables are expressed as frequency (n,%).

| Table S4 Correlations of studied variables | | | | | | | | | | | | | | | | | | |
| --- | --- | --- | --- | --- | --- | --- | --- | --- | --- | --- | --- | --- | --- | --- | --- | --- | --- | --- |
|  | 1 | 2 | 3 | 4 | 5 | 6 | 7 | 8 | 9 | 10 | 11 | 12 | 13 | 14 | 15 | 16 | 17 | 18 |
| 1.Child age | — |  |  |  |  |  |  |  |  |  |  |  |  |  |  |  |  |  |
| 2.Child gender | -0.017^*^ | — |  |  |  |  |  |  |  |  |  |  |  |  |  |  |  |  |
| 3.Primary parent age | 0.140^**^ | -0.017^*^ | — |  |  |  |  |  |  |  |  |  |  |  |  |  |  |  |
| 4.Primary parent gender | 0.016^*^ | 0.017^*^ | -0.166^**^ | — |  |  |  |  |  |  |  |  |  |  |  |  |  |  |
| 5.Smoking history | -0.019^**^ | -0.008 | 0.117^**^ | -0.722^**^ | — |  |  |  |  |  |  |  |  |  |  |  |  |  |
| 6.Alcohol history | 0.003 | -0.008 | 0.098^**^ | -0.611^**^ | 0.612^**^ | — |  |  |  |  |  |  |  |  |  |  |  |  |
| 7.Number of children | 0.099^**^ | 0.020^**^ | 0.297^**^ | 0.038^**^ | -0.035^**^ | -0.024^**^ | — |  |  |  |  |  |  |  |  |  |  |  |
| 8.Marital status | 0.030^**^ | 0.010 | -0.003 | -0.078^**^ | 0.089^**^ | 0.055^**^ | -0.049^**^ | — |  |  |  |  |  |  |  |  |  |  |
| 9.Employment status | 0.026^**^ | -0.003 | -0.077^**^ | 0.267^**^ | -0.200^**^ | -0.176^**^ | 0.168^**^ | -0.015^*^ | — |  |  |  |  |  |  |  |  |  |
| 10.Education level | -0.055^**^ | 0.007 | 0.045^**^ | -0.054^**^ | -0.028^**^ | 0.025^**^ | -0.231^**^ | -0.093^**^ | -0.430^**^ | — |  |  |  |  |  |  |  |  |
| 11.Annual family income | -0.038^**^ | -0.003 | 0.034^**^ | -0.060^**^ | 0.034^**^ | 0.059^**^ | -0.106^**^ | -0.078^**^ | -0.228^**^ | 0.302^**^ | — |  |  |  |  |  |  |  |
| 12.Household registration | -0.001 | -0.006 | -0.214^**^ | 0.053^**^ | -0.014^*^ | -0.050^**^ | 0.085^**^ | 0.031^**^ | 0.266^**^ | -0.432^**^ | -0.212^**^ | — |  |  |  |  |  |  |
| 13.Average daily screen time (weekdays) | 0.011 | -0.027^**^ | 0.007 | -0.027^**^ | 0.042^**^ | 0.066^**^ | 0.027^**^ | 0.014^*^ | 0.038^**^ | -0.079^**^ | -0.011 | 0.042^**^ | — |  |  |  |  |  |
| 14.Average daily screen time (weekends) | 0.021^**^ | -0.029^**^ | 0.018^**^ | -0.011 | 0.033^**^ | 0.058^**^ | 0.024^**^ | 0.011 | 0.037^**^ | -0.039^**^ | 0.039 | 0.026^**^ | 0.710^**^ | — |  |  |  |  |
| 15.Average daily screen time | 0.013 | -0.025^**^ | 0.005 | -0.022^**^ | 0.040^**^ | 0.062^**^ | 0.029 | 0.018^*^ | 0.045^**^ | -0.081^**^ | -0.004 | 0.044^**^ | 0.967^**^ | 0.835^**^ | — |  |  |  |
| 16.Total difficulties | -0.008 | -0.025^**^ | -0.029^**^ | -0.055^**^ | 0.051^**^ | 0.060^**^ | 0.006 | 0.031^**^ | 0.050^**^ | -0.092^**^ | -0.092^**^ | 0.052^**^ | 0.091^**^ | 0.057^**^ | 0.084^**^ | — |  |  |
| 17.Internalizing problems | 0.019^**^ | 0.017^*^ | -0.025^**^ | -0.048^**^ | 0.045^**^ | 0.043^**^ | 0.019^**^ | 0.032^**^ | 0.063^**^ | -0.088^**^ | -0.090^**^ | 0.049^**^ | 0.064^**^ | 0.038^**^ | 0.058^**^ | 0.657^**^ | — |  |
| 18.Externalizing problems | -0.030^**^ | -0.027^**^ | -0.020^**^ | -0.024^**^ | 0.020^**^ | 0.032^**^ | -0.014^*^ | 0.009 | 0.006 | -0.011 | -0.057^**^ | 0.005 | 0.068^**^ | 0.043^**^ | 0.062^**^ | 0.563^**^ | 0.375^**^ | — |
| 19.parental discipline | 0.024^**^ | -0.001^**^ | 0.021^**^ | -0.039^**^ | 0.0298^**^ | 0.024^**^ | 0.065^**^ | 0.009 | 0.011 | -0.062^**^ | -0.075^**^ | 0.035 | 0.084^**^ | 0.046^**^ | 0.070^**^ | 0.197^**^ | 0.163^**^ | 0.176^**^ |

Note: *Correlation significant at the .05 level (two-tailed). **Correlation significant at the 0.01 level (two-tailed).

Table S5 Summary of generalized additive model fit statistics for each outcome

| **Mental health outcomes** | **edf** | ***χ²*** | ***P*-value** |
| --- | --- | --- | --- |
| **Total difficulties** | 2.7093 | 131.2870 | <.001 |
| **Externalizing problems** | 2.3875 | 87.7583 | <.001 |
| **Internalizing problems** | 5.6175 | 75.2944 | <.001 |

Note: *χ²* values from generalized additive model chi-square tests for smooth terms.

Table S6 Threshold effect analysis of average daily screen time and total difficulties (TDS > 14)

| **Model** | **Value / OR (95% CI)** | ***P*-value** |
| --- | --- | --- |
| **One‑line linear** | 1.003 (1.002–1.004) | <.001 |
| **Two‑piece piecewise** |  |  |
| Break point, min | 70.71 (66.43–75.00) | <.001 |
| Below threshold (<70.71 min) |  |  |
| Per 1-minute increase | 1.01 (1.0063–1.0103) | <.001 |
| Per 60-minute increase (calculated) | 1.65 (1.46–1.85) | <.001 |
| Above threshold (>70.71 min) |  |  |
| Per 1-minute increase | 1.001 (1.000–1.002) | .014 |
| Per 60-minute increase (calculated) | 1.07 (1.01–1.13) | .014 |
| Log‑likelihood ratio test (two‑piece vs. one‑line) |  | <.001 |

Note: Hourly ORs were obtained by exponentiating the per‑minute log‑odds multiplied by 60; *P*‑values are identical due to the scale invariance of the Wald test.

Table S7. Model fit comparison: GAM vs. two‑piecewise logistic regression

| **Model** | **AIC** | **ΔAIC (GAM – two‑piecewise)** |
| --- | --- | --- |
| GAM | 26912.08 | Reference |
| Two‑piecewise | 26918.86 | −6.78 |

Note: AIC values can be large in magnitude due to the likelihood calculation for binary outcome models with N = 21,366, and should be interpreted in terms of relative differences rather than absolute values. The ΔAIC of −6.78 (favoring GAM) is well below the conventional threshold of 10, indicating comparable model fit between the two approaches.

Figure S1. Nonlinear associations between average daily screen time and total difficulties: binary and continuous outcomes. (A) Binary total difficulties (cutoff >14), (B) Continuous total difficulty scores. The red solid line represents the fitted curve, and the blue dashed lines represent the 95% confidence intervals. The vertical line in Panel A marks the policy-interpretable summary estimate (70 min/day, 95% CI 66–75) derived from two-piecewise logistic regression for clinical risk stratification; the vertical line in Panel B denotes the inflection point (92 min/day, 95% CI 75–113) from segmented linear regression representing symptom saturation.

Table S8 Summary of generalized additive model fit statistics for continuous total difficulty scores

| **Mental health outcomes** | **edf** | ***χ²*** | ***P*-value** |
| --- | --- | --- | --- |
| **Total difficulties** | 7.0128 | 45.7687 | <.001 |

Note: χ² values from generalized additive model chi-square tests for smooth terms.

Table S9 Threshold effect analysis of average daily screen time and continuous total difficulty scores

| **Model** | **Value / *β* (95% CI)** | ***P*-value** |
| --- | --- | --- |
| **One‑line linear** | 0.01 (0.01, 0.01) | <.001 |
| **Two‑piece piecewise** |  |  |
| Break point, min | 92.14 (75.00–113.17) | <.001 |
| Below threshold (<92.14 min) |  |  |
| Per 1-minute increase | 0.02 (0.02, 0.03) | <.001 |
| Above threshold (>92.14 min) |  |  |
| Per 1-minute increase | -0.00 (-0.00, 0.00) | .749 |
| Log‑likelihood ratio test (two‑piece vs. one‑line) |  | <.001 |

Table S10 Unadjusted crude multivariable logistic regression between screen time and mental health outcomes corresponding to Table 2 (N=21,366)

|  |  | **Total difficulties** | |  | **Externalizing problem** | |  | **Internalizing problem** | |
| --- | --- | --- | --- | --- | --- | --- | --- | --- | --- |
|  |  | OR (95%CI) | *P* value |  | OR (95%CI) | *P* value |  | OR (95%CI) | *P* value |
| **Average daily screen time (per 1-hour increase)** | | | | | | | | | |
|  |  | 1.22 (1.18–1.26) | <.001 |  | 1.15 (1.11–1.18) | <.001 |  | 1.15 (1.11–1.18) | <.001 |
| **Daily screen time (weekdays)** | | | | | | | | | |
| <30 min |  | 1.0 (ref) |  |  | 1.0 (ref) |  |  | 1.0 (ref) |  |
| 30 to 59 min |  | 1.28 (1.18–1.40) | <0.001 |  | 1.23 (1.14–1.32) | <.001 |  | 1.14 (1.05–1.23) | .002 |
| 60 to 119 min |  | 1.74 (1.56–1.94) | <.001 |  | 1.43 (1.30–1.58) | <.001 |  | 1.52 (1.37–1.69) | <.001 |
| 120 to 180 min |  | 1.95 (1.65–2.29) | <.001 |  | 1.63 (1.39–1.89) | <.001 |  | 1.58 (1.34–1.85) | <.001 |
| ＞ 180 min |  | 1.90 (1.69–2.12) | <.001 |  | 1.56 (1.41–1.74) | <.001 |  | 1.50 (1.34–1.67) | <.001 |
| **Daily screen time (weekends)** | | | | | | | | | |
| <30 min |  | 1.0 (ref) |  |  | 1.0 (ref) |  |  | 1.0 (ref) |  |
| 30 to 59 min |  | 0.94 (0.86–1.04) | .257 |  | 0.99 (0.91–1.09) | .870 |  | 0.89 (0.81–0.98) | .016 |
| 60 to 119 min |  | 1.01 (0.90–1.12) | .900 |  | 1.11 (1.01–1.22) | .039 |  | 0.97 (0.87–1.07) | .522 |
| 120 to 180 min |  | 1.26 (1.10–1.44) | <.001 |  | 1.22 (1.08–1.38) | .001 |  | 1.14 (1.00–1.30) | .045 |
| ＞ 180 min |  | 1.46 (1.31–1.63) | <.001 |  | 1.30 (1.18–1.44) | <.001 |  | 1.24 (1.12–1.38) | <.001 |

Note: Crude Model : Adjusted for none.

Table S11 Subscale-specific mediation effects of three dysfunctional parenting dimensions linking screen time to child mental health outcomes (N=21,366)

| Outcome variable | Parenting subscale | Total effect (95% CI) | Mediation effect (average)  (95% CI) | Direct effect (average) (95% CI) | Proportion mediated (average)  (95% CI) |
| --- | --- | --- | --- | --- | --- |
| Total difficulties | Over-reactivity | 0.023 (0.019–0.027) | 0.005 (0.004–0.006) | 0.018 (0.015–0.022) | 0.201 (0.154–0.259) |
|  | Laxness | 0.023 (0.019–0.026) | 0.001 (0.000–0.001) | 0.022 (0.018–0.026) | 0.030 (0.014–0.049) |
|  | Verbosity | 0.023 (0.019–0.026) | -0.000 (-0.001–-0.000) | 0.023 (0.019–0.027) | -0.018 (-0.033–-0.003) |
| Externalizing problem | Over-reactivity | 0.022 (0.017–0.026) | 0.005 (0.004–0.006) | 0.017 (0.012–0.021) | 0.230 (0.171–0.309) |
|  | Laxness | 0.021 (0.016–0.026) | 0.001 (0.000–0.001) | 0.021 (0.016–0.025) | 0.027 (0.012–0.044) |
|  | Verbosity | 0.021 (0.016–0.026) | -0.001 (-0.001–-0.000) | 0.022 (0.017–0.026) | -0.024 (-0.047–-0.005) |
| Internalizing problem | Over-reactivity | 0.016 (0.012–0.021) | 0.004 (0.003–0.005) | 0.013 (0.009–0.017) | 0.230 (0.166–0.322) |
|  | Laxness | 0.016 (0.012–0.021) | 0.001 (0.000–0.001) | 0.015 (0.011–0.020) | 0.040 (0.018–0.070) |
|  | Verbosity | 0.016 (0.012–0.021) | -0.000 (-0.001–-0.000) | 0.016 (0.012–0.021) | -0.021 (-0.045–-0.004) |

Table S12 Sensitivity analysis: Association between screen time dichotomized at 70.71 minutes and child mental health outcomes. (N=21,366)

|  |  | **Total difficulties** | |  | **Externalizing problem** | |  | **Internalizing problem** | |
| --- | --- | --- | --- | --- | --- | --- | --- | --- | --- |
|  |  | OR (95%CI) | *P* value |  | OR (95%CI) | *P* value |  | OR (95%CI) | *P* value |
| **Average daily screen time** | | | | | | | | | |
| ≤ 70.71 min |  | 1.0 (ref) |  |  | 1.0 (ref) |  |  | 1.0 (ref) |  |
| > 70.71 min |  | 1.48 (1.37–1.59) | <.001 |  | 1.31 (1.22–1.40) | <0.001 |  | 1.33 (1.24–1.43) | <.001 |

Note: Adjusted for child gender, child age, primary parent gender, primary parent age, smoke and alcohol use, number of children, marital status, education level, occupation, family income, and household registration of children.

Table S13 Sensitivity analysis: Association between screen time and child mental health outcomes excluding extreme screen time (≥180 min) participants. (N=18,953)

|  |  | **Total difficulties** | |  | **Externalizing problem** | |  | **Internalizing problem** | |
| --- | --- | --- | --- | --- | --- | --- | --- | --- | --- |
|  |  | OR (95%CI) | *P* value |  | OR (95%CI) | *P* value |  | OR (95%CI) | *P* value |
| **Daily screen time (weekdays)** | | | | | | | | | |
| <30 min |  | 1.0 (ref) |  |  | 1.0 (ref) |  |  | 1.0 (ref) |  |
| 30 to 59 min |  | 1.31 (1.20–1.43) | <.001 |  | 1.24 (1.15–1.34) | <.001 |  | 1.16 (1.07–1.26) | <.001 |
| 60 to 119 min |  | 1.75 (1.56–1.95) | <.001 |  | 1.44 (1.31–1.60) | <.001 |  | 1.54 (1.39–1.71) | <.001 |
| 120 to 180 min |  | 1.88 (1.59–2.22) | <.001 |  | 1.63 (1.40–1.91) | <.001 |  | 1.55 (1.32–1.82) | <.001 |

Note: Adjusted for child gender, child age, primary parent gender, primary parent age, smoke and alcohol use, number of children, marital status, education level, occupation, family income, and household registration of children .

Table S14 Sensitivity analysis: E-values for the association between high screen time (≥70.71 min/day) and mental health outcomes.

| **Outcome** | **Adjusted OR (95%CI)** | **RR (95% CI)** | **E-value (point)** | **E-value (CI lower)** |
| --- | --- | --- | --- | --- |
| Total difficulties | 1.48 (1.37–1.59) | 1.22 (1.17–1.26) | 1.73 | 1.62 |
| Externalizing problem | 1.31 (1.22–1.40) | 1.14 (1.10–1.18) | 1.55 | 1.44 |
| Internalizing problem | 1.33 (1.24–1.43) | 1.15 (1.11–1.20) | 1.57 | 1.47 |

Note: RR=risk ratio approximated from OR assuming outcome prevalence ≈18.6% (rare = FALSE).

Table S15 Sensitivity analysis: Multivariable logistic regression between screen time and mental health outcomes (pooled estimates from 5 imputed datasets)

|  |  | **Total difficulties** | |  | **Externalizing problem** | |  | **Internalizing problem** | |
| --- | --- | --- | --- | --- | --- | --- | --- | --- | --- |
|  |  | OR (95%CI) | *P* value |  | OR (95%CI) | *P* value |  | OR (95%CI) | *P* value |
| **Daily screen time (weekdays)** | | | | | | | | | |
| <30 min |  | 1.0 (ref) |  |  | 1.0 (ref) |  |  | 1.0 (ref) |  |
| 30 to 59 min |  | 1.22 (1.13–1.32) | <.001 |  | 1.18 (1.10–1.27) | <.001 |  | 1.12 (1.04–1.21) | .003 |
| 60 to 119 min |  | 1.62 (1.47–1.79) | <.001 |  | 1.39 (1.26–1.52) | <.001 |  | 1.45 (1.32–1.60) | <.001 |
| 120 to 180 min |  | 1.82 (1.57–2.11) | <.001 |  | 1.60 (1.39–1.84) | <.001 |  | 1.53 (1.33–1.77) | <.001 |
| ＞ 180 min |  | 1.69 (1.53–1.87) | <.001 |  | 1.49 (1.35–1.64) | <.001 |  | 1.37 (1.24–1.51) | <.001 |
| **Daily screen time (weekends)** | | | | | | | | | |
| <30 min |  | 1.0 (ref) |  |  | 1.0 (ref) |  |  | 1.0 (ref) |  |
| 30 to 59 min |  | 0.96 (0.88–1.05) | .366 |  | 0.97 (0.89–1.05) | .461 |  | 0.92 (0.85–1.01) | .067 |
| 60 to 119 min |  | 1.02 (0.92–1.13) | .6931 |  | 1.07 (0.98–1.17) | .142 |  | 0.99 (0.90–1.08) | .763 |
| 120 to 180 min |  | 1.24 (1.10–1.40) | <.001 |  | 1.20 (1.07–1.34) | .001 |  | 1.15 (1.02–1.29) | .020 |
| ＞ 180 min |  | 1.37 (1.24–1.51) | <.001 |  | 1.26 (1.15–1.38) | <.001 |  | 1.18 (1.07–1.30) | <.001 |

Note: Adjusted for child gender, child age, primary parent gender,primary parent age, smoke and alcohol use, number of children, marital status, education level, occupation, family income, and household registration of children.

Table S16 Sensitivity analysis: Reverse-direction mediation model (child mental health → parental discipline → screen time) (N=21,366)

|  | **Total difficulties** |  | **Externalizing problem** |  | **Internalizing problem** |
| --- | --- | --- | --- | --- | --- |
|  | Estimate (95%CI) |  | Estimate (95%CI) |  | Estimate (95%CI) |
| Total effect | 10.95 (8.89–13.05) |  | 8.07 (6.20–9.88) |  | 6.90 (5.02–8.91) |
| Mediation effect (average) | 1.53 (1.12–1.93) |  | 1.34 (0.99–1.68) |  | 1.32 (1.02–1.64) |
| Direct effect (average) | 9.42 (7.27–11.60) |  | 6.73 (4.81–8.59) |  | 5.57 (3.53–7.70) |
| Propotion mediated (average) | 0.139 (0.098–0.190) |  | 0.166 (0.115–0.238) |  | 0.192 (0.133–0.284) |

Note: Adjusted for child gender, child age, primary parent gender,primary parent age, smoke and alcohol use, number of children, marital status, education level, occupation, family income, and household registration of children .

Outcome variable (screen time) was modeled as a continuous variable (minutes/day); therefore, effect estimates are on the original scale and are not directly comparable to the forward model estimates, which were on the probit scale. The proportion mediated is a relative measure and is comparable across models.
